# Psychological, cognitive, and physiological impact of hazards casualties' trainings on first responders: the example of a chemical and radiological training. An exploratory study

**DOI:** 10.3389/fpsyg.2024.1336701

**Published:** 2024-01-30

**Authors:** Louise Giaume, Barbara Le Roy, Yann Daniel, Heloise Lauga Cami, Daniel Jost, Stéphane Travers, Marion Trousselard

**Affiliations:** ^1^Emergency Medical Department, Paris Fire Brigade, Paris, France; ^2^French Military Biomedical Research, Brétigny-sur-Orge, France; ^3^Centre National d'Etudes Spatiales, Paris, France; ^4^Medical School, University of Bordeaux, Bordeaux, France

**Keywords:** CBRN, simulation training, first responders, heart rate variability, parasympathetic system, stress, disaster medicine

## Abstract

**Background:**

First responders are among the first to respond to hazards casualties. They might operate in volatile, uncertain, complex, and ambiguous (VUCA) environments. While they have underlined the need to improve their knowledge and training to face these environments, there are few data regarding the stress induced by these trainings. Chemical, biological, radiological, and nuclear (CBRN) hazards casualties' trainings seem to be a good model of “*in vivo*” stress. First responders must operate in a hostile and encountered environment with a CBRN protective equipment that places demand on their psychological, cognitive, and physiological capacities. Current research recognizes that the activity of the parasympathetic system (PSS) can be used as an objective marker of stress adaptation, measured as heart rate variability (HRV).

**Objectives:**

To compare between baseline and simulation the evolution of the parasympathetic activity (primary outcome), anxiety, emotions, cognitive load, and body posture awareness (secondary outcomes).

**Methods:**

A total of 28 first responders attended to three simulated scenarios requiring CBRN management of casualties. One day before simulation, we collected HRV data (baseline). The simulations' day (pre-, post-simulation) we collected anxiety score (STAI-Y B), emotions (SPANE), cognitive load (NASA TLX), body posture awareness (PAS) and HRV. The morning after we collected the PAS score (recovery). We compare data' evolution between different times of the simulation.

**Results:**

(i) A high level of anxiety at baseline [*Median* 51 (46; 56)] which decreased between pre- and post-simulation (*p* = 0.04; *F* = 2.93); (ii) a post-simulation decrease in negative feelings (*p* = 0.03); (iii) a decrease in body awareness after simulation which returned to the initial level at recovery (*p* = 0.03; *F* = 3.48); (iv) a decrease in mean RR between baseline, pre- and post-simulation (*p* = 0.009; *F* = 5.11). There were no significant difference between times on others analysis of HRV.

**Conclusion:**

Prior to simulation, participants experienced anticipatory anxiety. Simulations training practiced regularly could be one way to combat anticipatory anxiety.

## 1 Background

### 1.1 The physiological and psychological cost of stress on first responders

As they are often the first to arrive to the scene of an emergency or disaster, first responders face most pressing challenges such as accidents, or hazards casualties (Turner et al., 2016). Occasionally, they might operate in extreme and changing environments that place demand on their psychological, cognitive and physiological capacities and expose them to a higher risk of occupational stress injuries (Antony et al., 2020).

The stress response, initially termed *heterostasis*, refers to a costly mode of functioning that can be contrasted with *homeostasis*, the organism's most economical mode (Delgado-Moreno et al., 2019; Billman, 2020). Each physio-psychological response to a challenge is shaped by the concomitant stress response and the biological scars of earlier responses to past stressful experiences. It involves the body's capacity to recover and return to homeostasis, after experiencing stress. On a physiological point of view, Selye describe three stages of the body's response to stress in the General Adaptation Syndrome (Selye, 1950): (i) The alarm stage: the sympathetic system is activated to release stress hormones like adrenaline to prepare the body for immediate action; (ii) the resistance stage: the body attempts to adapt and cope with the ongoing stressor. Hormone levels, especially cortisol, remain elevated to help the body maintain its resistance to the stressor; and (iii) the recovery stage: when the stressor stops, the parasympathetic system is activated to help the body' recovery. However, when a stressor is too intense or last too long, the body resources are depleted, and the individual enters in the exhaustion stage. In this stage, the physiological response is deficient, and the individual is chronically exposed to stress hormones. The exhaustion stage can cause health issues such as cardiovascular pathology, weakened immune system, digestive disorders, sleep disorders, and mental health disorders like anxiety and depression. Thus, the quality of recovery determines the quality of the stress response during the next exposure.

On a psychological point of view, according to Lazarus and Folkman (1984), stress occurs when an individual perceives a situation as being beyond his resources and potentially endangering his wellbeing. This process involves both the stressors and the personal resources. The stressors can be external (e.g., environment constraints, operational demands, shift schedules etc…) or internal (e.g., self-imposed expectations, negative thoughts, mind wandering etc…). Resources, on the other hand, encompass various aspects, such as physical and mental capabilities, social support, coping skills, personal traits like neuroticism (Schlatter et al., 2022) and are specific to each individual.

### 1.2 The impact of changing environment

Responses to environmental change are associated with changes in activity in the autonomic nervous system (ANS), and the enteric components of the visceral motor system that govern smooth muscle, heart muscle, and the glands (Critchley and Harrison, 2013). The neurovisceral integration model (Thayer and Siegle, 2002; Thayer et al., 2012; Smith et al., 2017) describes the role of the ANS in the regulation of emotions, behaviors, and adaptation, in interaction with the central autonomic nervous network (CAN). Environmental change is integrated and processed by CAN brain structures comprised of a set of cortical and subcortical regions (e.g., the hypothalamus, the insula, the prefrontal cortex, the amygdala), linked to parasympathetic activity. For example, the parasympathetic branch of the ANS promotes activation of the prefrontal cortex, which is itself involved in the regulation of emotions (specifically, the extinction of fear, *via* its role in inhibiting amygdala activity). Current theory emphasizes the bidirectional nature of the ANS. The activity of its sympathetic and parasympathetic branches is modulated to initiate physiological and behavioral changes adapted to the demands of the situation. Consequently, cardiac activity is thought to reflect not only muscular and electrophysiological functions, but also the regulatory balance of a higher-level complex neural network (Thayer et al., 2012). Regardless of whether these systems contribute to the maintenance of homeostasis, interindividual differences are found in healthy subjects. Specifically, the higher the basal parasympathetic activity, the more the individual can react to environmental stimuli, and, therefore, the more adaptable he is. Previous study argues that heart rate variability (HRV) may reflect vulnerability to stress and has a role in quantifying physiological elasticity and behavioral flexibility (Kim et al., 2018).

### 1.3 Enaction and the experience of the individual in their environment

The individual is transformed by interactions with the world; he gives “form to his environment and is at the same time shaped by it” (Varela et al., 2016). Enaction is mediated by both *interoception*, which informs the subject about their state of homeostasis, and *exteroception*, which informs them about the state of their environment (Chen et al., 2021). This sensory engagement modulates the stress response, by adjusting regulatory systems to optimize homeostasis, and maintain health. Together, interoception and exteroception reflect our internal representation of our body in relation to the environment (Suzuki et al., 2013). These perceptions actively contribute to the adaptive response of subjects in environments with physical and psychological constraints (Levit-Binnun and Golland, 2011). The individual's response relies on *sensory integration*, which refers to the process by which the brain receives a message through the senses and transforms it into an adaptive behavior. Body perception distortions have been mainly studied in clinical subjects, as subjects suffering from fibromyalgia (Viceconti et al., 2022). But less is known on the relationships between stress and body perception distortions in healthy subjects whereas experiments in isolates and/or confines and/or extrem and/or unusual suggest a relationship between the quality of body perception and representation and adaptation to the stressful environment (Le Roy et al., 2023). Adaptive behavior has been defined as the appropriate use of sensory inputs from the body's receptors and involves two main modalities: ensuring survival (modulation) and relating to the environment (discrimination) (Kilroy et al., 2019). Both functions are essential to the individual's adaptation to the environment, and are modulated by proprioceptive, interactive, and exteroceptive receptors, which make up the feedback loops involved in alliesthesia. Feedback loops link internal and external sensations, along with interactions between them, while receptors are closely linked to five major brain structures (spinal cord, the thalamus, the limbic system, the cortex, and the cerebellum) (Phelps and LeDoux, 2005). This dynamic process contributes to the stress response, and the maintenance of homeostasis.

### 1.4 Operating in VUCA environment: a challenge for first responders

Daily, first responders operate in changing environments. In the 90's, The United States Army War College used the acronym VUCA (e.g., Volatile, Uncertain, Complex and Ambigue) to refer to turbulent and unpredictable environments that could affect and weaken organizations (Barber, 1992). Volatility refers to the rapid and unpredictable changes that occur in the external environment; Uncertainty refers to hesitation, indecisiveness, and the need for organizations to be more flexible to changing circumstances; Complexity refers to the intricate and interconnected nature of challenges faced in the VUCA environment; and Ambiguity represents the lack of clarity and the existence of multiple interpretations or perspectives on a situation (Raio et al., 2022).

### 1.5 The example of chemical and radiological hazards

One of the hazard casualties that might face first responders are chemical, biological, radiological, or nuclear (CBRN) hazards casualties. CBRN hazards casualties are particularly challenging because their occurrence is rare (Ramesh and Kumar, 2010). Most first responders have never been exposed to real CBRN situations, their level of training and knowledge are often insufficient (Kako et al., 2018). They must face the risk of exposure to life-threatening toxic agents and wear personal protective equipment (PPE) to limit the risk of intoxication and contamination. Several studies report that the use of CBRN PPE is linked to significantly higher stress levels, fatigue, the risk of dehydration, somatic anxiety, and adverse reactions on physical health such as headache, skin disorder and pressure injuries (Swaminathan et al., 2022). The continued use of gloves and respiratory protection also compromises communication, slows down triage and other life-saving activities, and alters external sensory information (Goetz et al., 2011; Blacker et al., 2013; Gómez-Oliva et al., 2019; Merchan and Clemente-Suárez, 2020).

The side effects induced by the use of CBRN PPE can also interfere with the relationship between the first responder and the victim as negative contextual factors triggering nocebo effects in nursing practice and may compromise the patient's survival (Palese et al., 2019).

In highly demanding situation like CBRN hazards casualties, adequate training and preparation are essential to first responders for maintaining their knowledge and improving their performance. The literature highlights that simulation training can generate a high level of stress in participants (Rebera and Rafalowski, 2014; Bauer et al., 2016). In addition, training in an environment not regularly encountered like CBRN environment, and wearing a CBRN PPE with respiratory protection might further increase participants' stress levels during their simulation trainings. Data suggest that immersion in non-ecological sensory environments may have deleterious effects on physiological, psychological, and cognitive capacities and lead to inappropriate responses to stress (Le Roy et al., 2023).

A CBRN hazard casualty training with CBRN PPE seems to be a good model of *in vivo* stress to study the psychological, physiological, and cognitive impact on participants and to better understand the stress response under an example of VUCA environment.

### 1.6 Experimental context

The Paris Fire Brigade (PFB) is the fire department of the City of Paris. It is a military institution that provides fire protection services, technical rescue/special operations services, chemical, biological, radiological, nuclear, and high-yield explosive/hazardous materials response services, and emergency medical response services within Paris and three adjacent departments (Seine Saint-Denis, Val-de-Marne, Hautes-Seine). All year round, 24 h a day, the PFB deploys six ALS ambulances each staffed by an emergency physician, a nurse, and an ambulance operator. These ambulances form part of the Parisian prehospital rescue system, along with the civilian emergency medical service (called SAMU), serving an area of 762 km^2^ and 7 million inhabitants, and receiving at the 1–1-2 call-center (the public access number for emergency services in France) 3,500 emergency calls per day. These ambulances are specialized in emergency medicine, disaster medicine, management of mass casualties, and CBRN hazards. In case of a CBRN attack in Paris and departments next door, they will be among the first to respond to manage CBRN casualties (SGDSN ciculaire n°800, 2011; SGDSN circulaire n°700, 2018).

Whether it is terrorist attack (Tin and Ciottone, 2022), war (the nuclear power plant of Zaporijia in Ukraine targeted by Russian strikes in July 2022), pandemics such as Ebola (Hampton et al., 2023) or COVID-19 (Sachs et al., 2022), current events are a constant reminder that the CBRN threat is always present. Previous studies have focused on fatigue, cognitive, psychological, and physiological stress response in participants wearing PPE compared to control group with participants without equipment in the military (Merchan and Clemente-Suárez, 2020). In this study, the use of PPE produced significantly higher stress, fatigue, temperature, heart rate and somatic anxiety compared to the participants without PPE. Other studies focused only on the physical reactions of the wear on PPE (Jiang et al., 2020; Lin et al., 2020; Zuo et al., 2020).

Based on these findings, we decided to conduct an exploratory study on the evolution of psychological, cognitive and physiological parameters in CBRN PPE during CBNR hazards casualties' management training in first responders for a description of the stress response in an enactive framework.

Our primary aim was to compare between baseline and simulation day (pre- and post-simulation), the evolution of the parasympathetic activity measured by the RMSSD in order to evaluate the physiological stress response using the consensual biomarker of stress adaptation.

Our secondary aims focused the psychological simulation's response. They were to compare (i) during simulation day the evolution of the anxiety score measured by the STAI-Y B questionnaire, positive and negative feelings measured by the SPANE, and cognitive load measured by the NASA TLX score; and (ii) during simulation day and recovery, the evolution of the body posture awareness by PAS score. An exploratory aim focused on the ANS response to the NRBC simulation training and compared between baseline and simulation day the evolution of HRV features analysis such as *time domain analysis*: mean RR, the standard deviation of the normal-to-normal RR interval (SDNN), the percentage of adjacent NN intervals that differ from each other by more than 50 ms (pNN50), *frequency analysis*: low frequency (LF) high frequency (HF); and *non-linear analysis*: the standard deviation of the instantaneous variability of the interbeat interval (SD1), the standard deviation of the long-term continuous variability of RR (SD2), α1, α2, and sample entropy (the regularity and complexity of the time series).

Our hypothesis was that we might observe at the end of the trainings:

- A decreased RMSSD (primary outcome).
- A degraded psychological functioning (i.e., increased anxiety, increased negative feelings).
- Acognitive overload.
- A decreased attention to body awareness (secondary outcomes).
- Changes in HRV responses due to the stressful training situation (exploratory outcomes) induced by simulation training.

## 2 Methods

### 2.1 Design

This pragmatic, exploratory, interventional, low-risk quasi experimental study was conducted with members of the ALS ambulance staff of the PFB, during three successive simulation trainings in chemical and radiological hazard casualties' management.

Each emergency physician, nurse, and ambulance operator of PFB' ALS ambulance staff attend every 2 years to a morning session of three simulation trainings on CRN hazard casualties to manage.

During the simulation training, participants must wear CBRN PPE and respiratory protection. Their task is to manage casualties who have been exposed to factice chemical or radiological agents, and to deploy tactical and therapeutic measures in three, increasingly stressful scenarios: (i) two victims contaminated by a neurotoxic agent; (ii) three victims caused by a dirty bomb with a radio contaminated agent; and (iii) a hostage situation with neurotoxic agent poisoning, and six victims to be treated.

Our study was submitted to the Committee of the Protection of Persons (RIPH2; ID- 2021-A03057–34) Ile de France XI, and received a favorable opinion on November 10, 2022

### 2.2 Participants

Inclusion criteria were: (i) aged over 18; (ii) being a member of the PFB' ALS ambulance staff; (iii) participating in the simulation training of a CBRN hazard casualty management; and (iv) a recent medical checkup. Exclusion criteria were: (i) refusal to participate in the study; (ii) a heart rhythm disorder; (iii) endocrine pathology (e.g., hyperthyroidism, diabetes); (iv) arterial hypertension; (v) current pregnancy or breastfeeding; (vi) ongoing anti-inflammatory treatment; (vii) ongoing treatment that may affect the heart rate (e.g., beta-blockers, calcium channel blockers, alpha-1 agonists); and (viii) psychotropic treatments. Secondary exclusion criteria were: (i) incomplete psychological and cognitive data, and low Cronbach's alpha; (ii) premature termination of the simulation exercise; and (iii) a poor-quality cardiac bio signal (e.g., artifacts, extrasystoles).

### 2.3 Measures

A 28-item socio-biographical questionnaire was used to collect standard sociodemographic data such as age, health status, and profession.

#### 2.3.1 Psychological measures

The following psychological measures were used.

The 12-item Postural Awareness Scale (PAS) assesses body posture awareness as two sub-factors, “ease/ familiarity with postural awareness” and “need for attention regulation with postural awareness” (Da Costa Silva et al., 2022). The 12-item Scale of Positive and Negative Experience (SPANE) assesses positive and negative affect (Diener et al., 2010). The State Anxiety Inventory (STAI Y-B) assesses the level of situational anxiety (Spielberger et al., 1983).

#### 2.3.2 Cognitive measures

The 6-item NASA Task Load Index was used to assess subjective mental load (Hart and Staveland, 1988).

#### 2.3.3 Heart rate variability

HRV was measured using heartbeat interval data recorded over a 10-min period using an electrocardiogram (EKG), with the BIOPAC system (MP35 Systems; CE; 1 kHz sampling rate) and three electrodes. The HRV analysis followed guidelines (Malik, 1996; Laborde et al., 2017). Circadian variation was accounted for using the PyHRV python library (Gomes et al., 2019). The following data were also recorded: weight, height, BMI, IMG, smoking habits, most recent alcohol intake (>24 h), most recent caffeine (coffee/ tea) consumption (>1 h), most recent meal (>2 h), most recent physical activity (>12 h), and sleep quality on the day of the simulation exercise, and the previous day. Raw EKG data were filtered between 3 Hz and 45 Hz using a finite impulse response bandpass filter. The filter order was set to 300 (0.3 times the sampling rate). R-peaks were automatically detected using the BioSPPy python library (Carreiras et al., 2015). Once the signal had been filtered, a Hamilton segmentation was performed, followed by an R-peak correction with a tolerance set to 0.05. The validity of the R-wave detection was manually examined to ensure correct detection. If an EKG sequence was too noisy when viewing the superposition of all QRS complexes, a time interval was manually removed to improve data quality. RR intervals were detected automatically, with the HRV analysis module using linear interpolation, and corrected manually for artifacts and ectopic beats. In the case of outliers, the RR intervals considered as correct were manually modified.

▪ *Time domain analysis*: time-domain HRV measurements included: mean RR (the mean interbeat interval); SDNN, which is composed of both sympathetic and parasympathetic activity; RMSSD, which reflects parasympathetic activity; and pNN50.
▪ *Frequency analysis*: frequency-domain HRV measurements complemented time-domain measurements and included the properties of oscillatory components in heart rate dynamics. Spectral density was estimated according to Welch's method: LF sympathetic activity was in the range 0.04 Hz−0.15 Hz, and HF parasympathetic activity was in the range 0.15 Hz−0.4 Hz.
▪ *Nonlinear analysis*: non-linear HRV indices reflect dynamic and chaotic internal states that other metrics cannot capture. We used the most representative ones: the Pointcaré plot (a graphical representation of the correlation between successive interbeat intervals); SD1 is associated with a parasympathetic activity; SD2 is associated with sympathetic activity; α1 (the self-similarity parameter that represents short-term fluctuations); α2 (the time-series self-similarity parameter that represents long-term fluctuations); and sample entropy (the regularity and complexity of the time series).

### 2.4 Data collection

The study was presented to participants. They signed the clinical consent form, inclusion and exclusion criteria were checked.

Data were collected at 3 different times:

- *HRV baseline data* were collected a day before the simulation training: HRV data were recorded for 10 min, participants were asked to sit with their knees bent and their palms pointing upwards at rest. The HRV recording took place in the afternoon, 2 h after lunch.
- Simulation training data were collected:

(i) *Pre-simulation* data were collected just before the three simulation trainings: body awareness (PAS), emotions (SPANE), anxiety (STAI-Y B) and cognitive load (NASA TLX) questionnaires were collected via WEPI with an internet link sent to the participants. HRV data were recorded for 10 min without the PPE and the respiratory protection. Participants were asked to sit with their knees bent and their palms pointing upwards at rest.
(ii) In *between simulation* 1 data (i.e., S1) and in between simulation 2 data (i.e., S2) were collected respectively after the first simulation and the second simulation: body awareness posture (PAS), anxiety (STAI-Y B) and cognitive load (NASA TLX) questionnaires were collected via WEPI with an internet link sent to the participants.
(iii) *Post-simulation data* were collected at the end of the three-simulation training: body awareness posture (PAS), anxiety (STAI-Y B), emotions (SPANE) and cognitive load (NASA TLX) questionnaires were collected via WEPI with an internet link sent to the participants. HRV data were recorded 15 min after the end of the last simulation, for 10 min without the PPE and the respiratory protection. Participants were asked to sit with their knees bent and their palms pointing upwards at rest.

- *Recovery data* were collected the morning after the simulation training: body awareness (PAS) questionnaire was collected via WEPI with an internet link sent to the participants.

### 2.5 Data analysis

On the basis of data from Shaffer and Ginsberg (2017) we can estimate the mean RMSDD score at 42, with a standard deviation of 10. Given that a 32% reduction in post-simulation would be relevant in view of our study on first responders, with a first-species risk set at 5% and power set at 90%, on a two-sided assumption, the number of subjects required for the study was 26 (Shaffer and Ginsberg, 2017).

Data were analyzed using Python (Python Software Foundation, Wilmington, v3.8) and JASP (JASP, Amsterdam, v 0.16.2) with respect to the stress response, the homoscedasticity of variance and the normality of the distribution were confirmed, justifying the use of parametric ANOVA. When these criteria were not met, non-parametric tests were used. For all comparisons, a *p*-value of ≤ 0.05 was considered statistically significant. A trend was considered when 0.05 < *p* ≤ 0.1.

## 3 Results

Twenty-nine members of the ALS ambulance team were volunteers to participate to the study. All were in good health. One participant was excluded, for medical reasons, during the experiment, leaving 28 participants (six women and 22 men, *Mage* = 32.62 ± 6.7 years, range 21–53). Ten people (35.71%) were driver operators, seven (25%) were nurses, and 12 (42.85%) were emergency physicians. Of the six women, four (14.81%) were using contraception (i.e., the pill, a copper intrauterine device). None were taking any medication. Five (17.85%) were smokers, and one (3.57%) had suffered a pneumothorax. Nine (33.33%) declared having previous experience of a major stressful life event. Ten (37.03%) reported occasional use of a stress management technique (i.e., meditation, self-hypnosis, cardiac coherence, autonomous sensory meridian response). Mean height = 175.00 ± 0.00 meters, and Mean weight *t* = 75.51 ± 13.85 kilos. Due to operational constraints, only 17 participants took part in all three simulations, while the remaining nine took part in two simulations.

### 3.1 Impact of the simulations on physiological, psychological, and cognitive

*Physiological data:* HRV data are showed in Table 1.

**Table 1:** HRV data on baseline, pre-simulation, and post simulation.

**HRV data *(Mean ±SD)***	**Baseline**	**Pre-simulation**	**Post-simulation**	***p***	***F***
**Time domain analysis**					
RMSSD (ms)	51.8 ± 28.8	47.4 ± 25.4	45.8 ± 26.3	0.36	1.02
Mean RR	957.4 ± 147	898.4 ± 119	881.3 ± 129	*0.009*	5.11
SDDN (ms)	73.9 ± 31.4	75.5 ± 29.9	68.9 ± 25.9	0.39	0.95
pNN50 (%)	31 ± 23.3	24.8 ± 20.6	24.3 ±21.4	0.2	1.65
**Frequency analysis**					
LF (ms)	2931.4 ± 2948.9	3160.7 ± 3206	3004 ± 3539	0.88	0.12
HF (ms)	1165.4 ± 1383.1	928.1 ± 1041	845 ± 975.2	0.26	1.3
**Non-linear analysis**					
SD1	36.6 ± 20.3	33.5 ± 17.9	32.4 ± 18.6	0.3	1.02
SD2	97 ± 40.9	100.5 ± 40	90.88 ± 3.9	0.34	1.07
α1	1.06 ± 0.3	1.19 ± 0.3	1.2 ± 0.3	*0.008*	5.2
α2	0.75 ± 0.26	0.81 ± 0.31	0.72 ± 0.28	*0.03*	3.45
Sample entropy	1.32 ± 0.31	1.26 ± 0.34	1.27 ± 0.38	0.4	0.75

A p ≤ 0.05 was considered statistically significant. SD, standard deviation.

There was no significant difference on RMSDD between baseline, pre- and post-simulation (*p* = 0.36; *F* = 1.02) (primary outcome).

**Figure 1:**
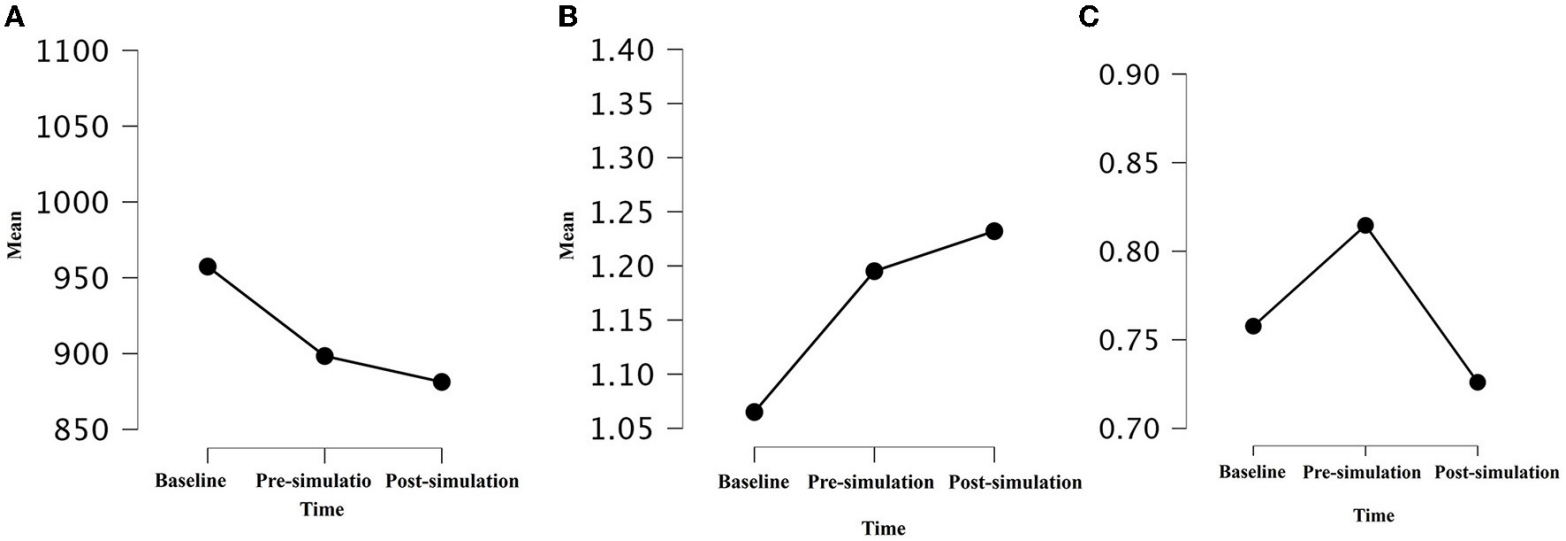
Mean of Mean RR **(A)**, α1 **(B)**, and α2 **(C)** during baseline, pre- and post-simulation (*p* = 0.009, *F* = 5.11; *p* = 0.008, *F* = 5.2; *p* = 0.03, *F* = 3.45). A *p-*value of ≤ 0.05 was considered statistically significant.

There was a significant decrease in mean RR between baseline, pre- and post-simulation (*p* = 0.009; *F* = 5.11) as showed in Figure 1A; an increase in α1 between baseline, pre- and post-simulation (*p* = 0.008; *F* = 5.2) as showed in Figure 1B; and an increase in α2 between baseline and pre-simulation followed by a decrease between pre- and post-simulation (*p* = 0.03; *F* = 3.45) as showed in Figure 1C.

The rest of the HRV analysis were non-significant between times.

#### 3.1.1 Psychological measures

**Figure 2:**
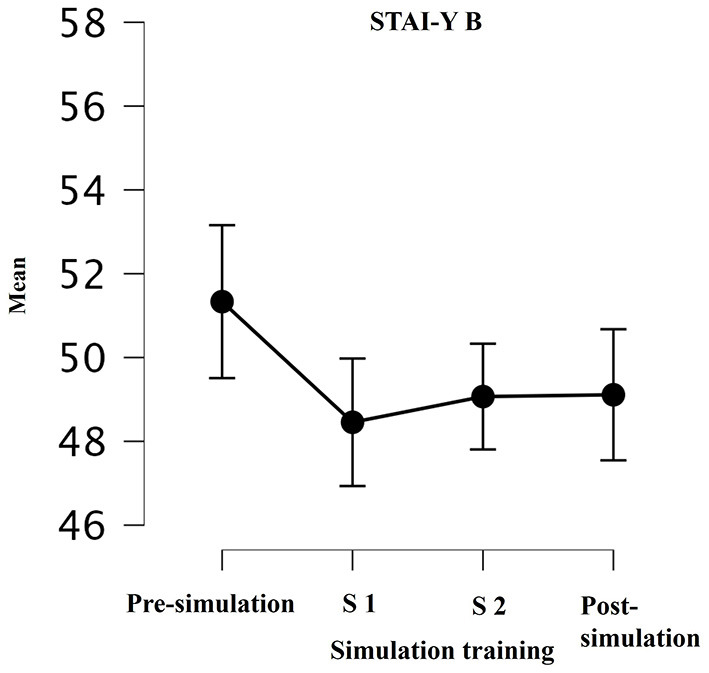
Mean of STAY-I during simulation training: pre-training; in between simulation 1 (S1); in between simulation 2 (S2); post-training (*p* = 0.02; *p* = 0.04). A *p-*value of ≤ 0.05 was considered statistically significant.

*State anxiety* (STAI Y-B): there was a significant decrease in state anxiety between pre- and post-simulation measures in subjects who took part in all three simulations as showed in Figure 2 [pre-simulation: 51.3 ± 2.7 (*M* ± *SD*); in between simulation 1: 48.4 ± 4.3 (*M* ± *SD*); in between simulation 2: 49 ± 3.95 (*M* ± *SD*); post-simulation: 49.1 ± 4.6 (*M* ± *SD*); *p* = 0.04; *F* = 2.93]. However, this difference was not observed in subjects who only completed two simulations (*p* = 0.25; *F* = 1).

*Positive and negative affect* (SPANE): there was a significant decrease in negative affect between pre- and post-simulation measures (pre-simulation: 1.8 ± 0.5 (*M* ± *SD*); post-simulation 1.7 ±0.42 (*M* ± *SD*); *p* = 0.03). There was no significant effect on positive feeling between pre- and post-simulation (*p* = 0.5)

#### 3.1.2 Cognitive measures

There was no significant difference in cognitive load between pre- and post-simulation, measures, either in subjects who participated in two (*p* = 0.5), or three simulations (*p* = 0.3; *F* = 1.07).

#### 3.1.3 Body awareness (PAS)

**Figure 3:**
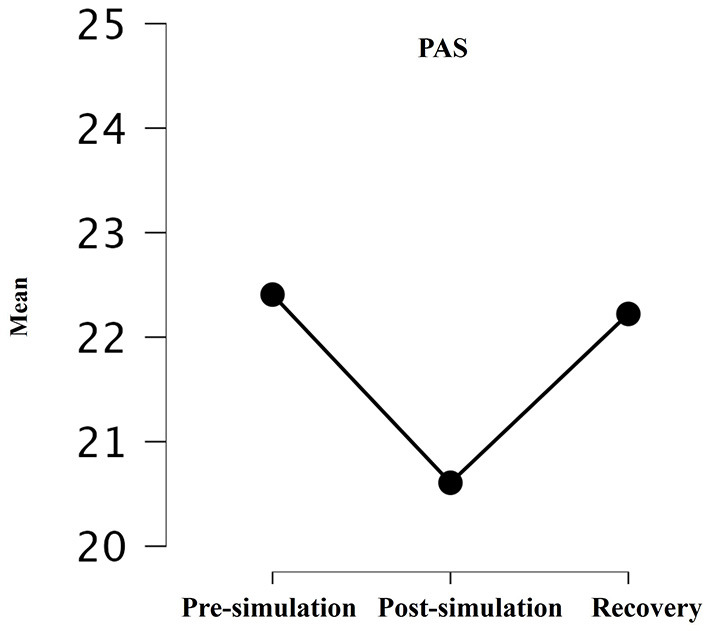
Mean of PAS pre-training, post-training, and recovery *p* = 0.06; *p* = 0.07. A *p*-value of ≤ 0.05 was considered statistically significant; A trend was considered when 0.05 < *p* ≤ 0.1.

A significative time effect was observed with a decrease observed for ease and body awareness between pre- and post-simulation, along with a return to the initial level between post-simulation and recovery [pre-simulation: 22.4 ± 7.1 (*M* ± *SD*); post simulation: 20.6 ± 7.5 (*M* ± *SD*); recovery: 22.2 ± 7.8(*M* ± *SD*); *p* = 0.038; *F* = 3.48] as shown in Figure 3.

## 4 Discussion

Our results confirm that CRN hazard casualties' simulation have an impact on psychological and physiological first responders' capacities, particularly prior the simulation. Indeed, state anxiety (measured using the STAI-Y scale), decreased significantly between pre-simulation and the first simulation, before stabilizing during the following simulations, and negative feelings decreased significantly between pre- and post-simulation. These psychological findings seem to be confirmed by the HRV, as mean RR decreased significantly between baseline, pre- and post-simulation. The time prior the simulation training seems to be a vulnerable moment that may generate anticipatory anxiety in participants. Anticipatory anxiety is frequently observed in simulation trainings, as participants may feel that they will be observed and judged by their peers, generating additional stress (Schlatter et al., 2022). The phenomenon has also been described in skydivers, with an increase anxiety before a skydive (Clemente-Suárez et al., 2017).

In terms of subjective proprioception, we observed a significant decrease on body awareness between pre- and post-simulation, that return to the initial level at recovery, 24 h later. Thus, this alteration seems reversible and subject to further exploration, it is reasonable to think that a prolonged exercise may have an impact on body postural awareness and on its recovery and to suggest that proprioceptive functioning may be decreased. Emerging reviews show evidence that the SNA, the sympathetic division in particular, routinely participates in postural control (Sibley et al., 2014).

Given all these observations and the experimental context with the wear of CBRN PPE, we were surprised to find no other significant modification on HRV variables, and especially on RMSSD between baseline, pre- and post-simulation. Furthermore, the non-linear HRV variables did not change, except the α1 and α2. Short terms fluctuations increased during baseline and pre-simulation whereas long terms fluctuations increased during pre- and post-simulation. This suggests that self-similarity HRV was impact by the simulation.

Studies suggest that human adaptation to a stressful environment may rely heavily on the functioning of the interoceptive network, which provides moment-by-moment awareness (Pinna and Edwards, 2020). Regardless of how exteroception and interoception interact to guide adaptation, the individual's interoceptive capacity is thought to shape their ability to respond to external stimuli in the long term by orchestrating regulatory responses at both the conscious level, through emotions and feelings, and the autonomic level (Chen et al., 2021). Moreover, the link between HRV and interoceptive awareness is becoming increasingly evident (De Witte et al., 2016). Higher HRV, measured as RMSSD, and better interoception have been associated with better emotional regulation, and better-adjusted behavioral responses. Increased anxiety, rumination, and difficulties in controlling emotions are associated with a decrease HRV (Thayer et al., 2012).

We were also surprised to observe no significant differences in the cognitive load between the three CBRN hazard casualties' scenarios, while their complexity increased.

Based on these findings, we expected to observe changes in HRV, especially in RMSSD, between baseline and simulation like we observed with the psychological and proprioceptive measures. This lack of consistency between physiological, cognitive and psychological measures could indicate that these variables do not capture the same concept. Multiple levels of stress response may explain this finding. In practice, self-reported stress measures, and physiological stress outcomes are measured using two different assessment scales: self-administered questionnaires use a Likert-type scale with discrete intervals, while physiological outcomes are monitored continuously, and, most often, non-linearly (Epel et al., 2018).

## 5 Limitations

Our study shows several limitations. First, our small sample (28 subjects) lacks statistical power, which could explain the lack of significant results regarding psychological and cognitive measures.

Secondly, we did not compare our results with a control group. A control group would have enabled us to distinguish whether the physiological and psychological disturbances were linked to wearing the PPE. With the resources available, a control group was either an ethical or an experimental issue to us. If we set up a control group under the same training conditions (CBRN hazards casualties) without CBRN PPE, we risked training first responders without the right clinical habits. If we had set up a control group in another hazard casualties that did not require the wear of PPE, the experimental context would have been too different from the specificity of a CBRN hazard casualties. Our operational resources did not allow us to carry out several training sessions with and without the mask, and the goal of our institution is to train first responders correctly according to the guidelines of CBRN hazards casualties.

Another limitation relates to the experimental settings. The chemical and radiological environment was reproduced by scenarios where participants wear CBRN PPE and were exposed to hypothetical radiological and chemical agents and not odorants or real CBRN agents. This implies that first our experimental settings might underestimate the results that we would have been observed with real chemical or radiological agents. With real CBRN agents, we might observe higher score of anxiety in first responders that would have a significant effect on their behavioral and physiological stress response. And, secondly, we did not verify the airtightness of the respiratory protection. Therefore, the participants might not comply respect the strict PPE instructions wearing.

Also, the duration of the simulation trainings (i.e., one morning), might also not be insufficient to observe any significant effects. During the COVID-19 pandemic, the literature report that prolonged duration of PPE use improve the risk of device-related pressure injury (De Coelho et al., 2020; Jiang et al., 2020; Aksoy and Büyükbayram, 2022).

Also, we did not evaluate performance during the simulations, we cannot assess any operational repercussions.

Finally, we did not conduct a longitudinal study due to operational constraints. After training, most participants return to their operational shift and follow-up is complicated. Indeed, recovery HRV measures could have been relevant for better describe impacts on simulation training on HRV stress response as suggest in the vagal tank theory (Laborde et al., 2017).

However, we did not find any difference in terms of HRV, the anxiety level experienced by the participants before and during the simulation may have an impact on the learning process and the effectiveness of training in simulation (Foronda et al., 2013) according to the participants' psychological profiles (Stein, 2022).

It is important to take this stress into account to adapt the learning process so that participants can benefit as much as possible from simulation trainings. Practitioners generally show higher performance when they practice regularly (Lund-Kordahl et al., 2019). The lack of CBRN experience of first responders is likely to generate additional anticipatory anxiety to the anxiety classically generated by other hazard casualty situations (Kako et al., 2018). If the CBRN hazard casualties lasted longer, anticipatory anxiety could have a deleterious effect on the first responders' physiological and cognitive capacities. This additional component of the CBRN environment needs to be considered in the training. Simulation training based on critical situations or hazard casualties seems to have positive and rapid effects on stress and anxiety, and should be practiced regularly by first responders to reduce anticipatory anxiety (Couarraze et al., 2023).

During the EBOLA epidemic, and then the COVID-19 pandemic, healthcare worker (HCW) caring for infected patients in high risk-clinical setting have been obliged to wear PPE to limit the risk of exposure to toxic agents (Fischer et al., 2015; Tabah et al., 2020). On the mental wellbeing impact of wearing during these pandemics, studies report high score of somnolence, anxiety, insomnia, depression and fear of contracting the virus (Swaminathan et al., 2022; Vivion et al., 2023). At a time when the world is talking about the possibility of facing new CBRN attacks or new pandemics (Frieden et al., 2021), it seems essential to take into account the physiological, psychological and cognitive costs for the HCW.

## 6 Conclusion

Our preliminary findings offer a better understanding of the psychological, cognitive, and physiological functioning of first responders under “*in vivo* stress” conditions, and demonstrates that prior to CBRN hazards casualties' simulation training, participants experienced anticipatory anxiety. If the CBRN hazard casualties lasted longer, anticipatory anxiety could have a deleterious effect on the first responders' physiological and cognitive capacities that could increase the risk of errors with consequence for both victims and first responders. Further studies recorded over a longer period, with strict control of CBRN PPE tightness, and with real CBRN agents should be pursued to better understand the impact of anticipatory anxiety in the stress response during CBRN simulation trainings. First responders should practice simulations training regularly to combat anticipatory anxiety.

## Data availability statement

The raw data supporting the conclusions of this article will be made available by the authors, without undue reservation.

## Ethics statement

The studies involving humans were approved by Committee of the Protection of Person Ile de France XI RIPH2; ID- 2021-A03057–34. The studies were conducted in accordance with the local legislation and institutional requirements. The participants provided their written informed consent to participate in this study.

## Author contributions

LG: Conceptualization, Data curation, Formal analysis, Funding acquisition, Investigation, Methodology, Project administration, Resources, Supervision, Writing – original draft. BL: Conceptualization, Data curation, Formal analysis, Investigation, Methodology, Resources, Writing – review & editing. YD: Validation, Writing – review & editing. HL: Funding acquisition, Investigation, Writing – review & editing. DJ: Project administration, Validation, Writing – review & editing. ST: Validation, Writing – review & editing. MT: Supervision, Validation, Writing – review & editing.
