# Supplementary figures and images for: Psychological, cognitive, and physiological impact of hazards casualties' trainings on first responders: the example of a chemical and radiological training. An exploratory study
Source: Front Psychol. 2024 Jan 30;15:1336701. doi: 10.3389/fpsyg.2024.1336701 (PMC10861781; doi:10.3389/fpsyg.2024.1336701)

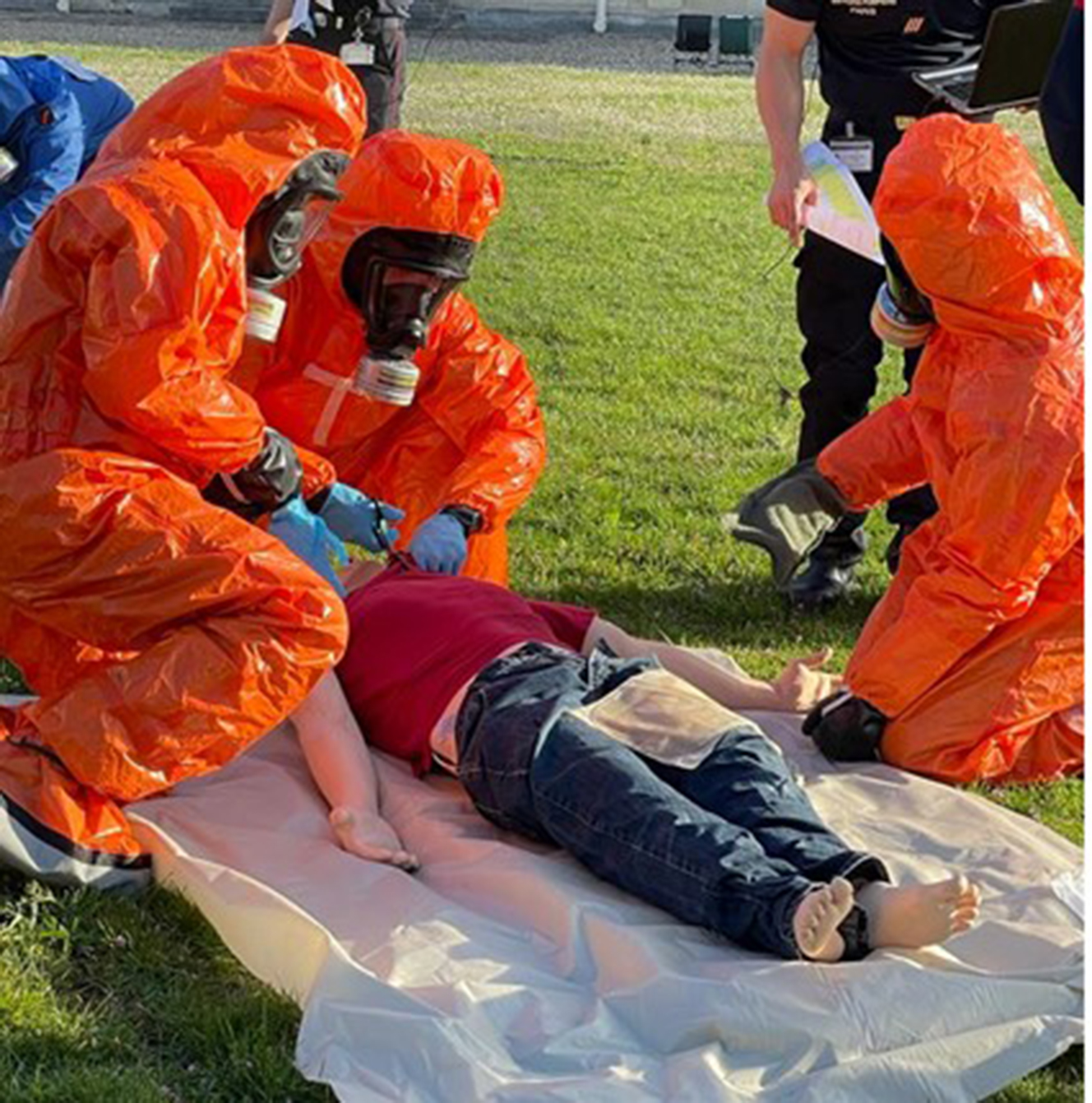

Supplement: Supplementary file 1 [file Image_1.JPEG]
